# Perspectives of ESCAPE-Pain Programme for Older People With Knee Osteoarthritis in the Community Setting

**DOI:** 10.3389/fpubh.2020.612413

**Published:** 2021-01-20

**Authors:** Muhammad Kamil Che Hasan, Emma Stanmore, Chris Todd

**Affiliations:** ^1^Kulliyyah (Faculty) of Nursing, International Islamic University Malaysia, Kuantan, Malaysia; ^2^School of Health Sciences and Manchester Academic Health Science Centre (MAHSC), Jean McFarlane Building, University Place, University of Manchester, Manchester, United Kingdom; ^3^Central Manchester University Hospitals NHS Foundation Trust, Manchester, United Kingdom

**Keywords:** osteoarthritis, exercise, self-management, primary care, community

## Abstract

**Background:** Functional limitationscommonly affect patients with knee osteoarthritis (OA) which reduces quality of life. The Enabling Self-management and Coping with Arthritic Pain using Exercise (ESCAPE-pain) is an evidence-based programme identified to be suitable for adaptation for the Malaysian health care system. It is important to understand the acceptance from a sociocultural context of the ESCAPE-pain programme from the perspectives of patients with knee OA and healthcare professionals. This qualitative study aims to explore the perspectives of stakeholders to inform the adaptation of the ESCAPE-pain programme into the Malaysian health care system.

**Method:** Semi-structured interviews using interview guides were conducted with 18 patients with knee OA and 14 healthcare professionals including nurses, physiotherapists, occupational therapists, medical doctors, and orthopedic surgeons. The data were transcribed and analyzed using framework analysis.

**Results:** The findings show that patients and healthcare professionals positively accept the programme into their daily living activities and recommend some modifications related to the Malaysian context. This study also highlights strategies to adopt when providing ESCAPE-pain to patients with knee OA.

**Conclusion:** The findings reveal how sociocultural considerations could facilitate uptake and engagement with the ESCAPE-pain programme for home exercise among patients with knee osteoarthritis. These findings may benefit t patients with knee OA in the Malaysian healthcare system, although future research is recommended.

## Introduction

Over the last 20 years, symptomatic knee OA has increased globally and it is anticipated that it will continue to increase due to growing levels of obesity and population survival into older age (1, 2). This requires healthcare professionals to prepare for the increased demand for services to treat OA (3). A study on the global burden of disease in 2012 estimated that 250 million people are affected by knee OA, with rates in females being almost double that in males worldwide (3, 4).

In Malaysia, the Arthritis Foundation of Malaysia (AFM) estimates that around one in ten older people aged 60 and above have OA, with the most common form being OA of the knee (5). Although there are no precise data for patients with knee OA currently in Malaysia, the Community Orientated Program for Control of Rheumatic Diseases (COPCORD) study on musculoskeletal pain concluded that the knee was responsible for the majority of all reported complaints of joint pain during the study, and on further examination more than half of those with knee pain had OA (6).

Current clinical practice guidelines (CPG) provided by the Malaysian Ministry of Health (MOH) for managing patients with knee OA focus on hospital-based care, which involves multidisciplinary approaches with pharmacological treatment and surgery alongside health education (7). It is recommended that patients with OA are educated about the diagnosis, weight reduction (for obese patients), range of motion exercise, strengthening, and aerobic exercise (7). Little is known about the strategies and delivery system used to ensure that the patients adhere to their prescribed treatment in Malaysia. It is also observed that no community or home-based programme for patients with knee OA are offered or implemented in the country as an alternative if they are unable to afford hospital treatment.

In order to explore how patients with OA can benefit from a self-management programme, we systematically searched the most feasible, evidence-based programme for OA. The ESCAPE-pain programme has been shown to be safe and cost-effective for individuals with either knee or hip OA (8–10). ESCAPE-pain is a rehabilitation programme that integrates educational self-management and coping strategies for people with joint pain with an individualized exercise regimen for each participant. The programme is available online at www.escape-pain.org to facilitate wider clinical implementation to benefit more people (11). In brief, the programme comprises two components, which are education and exercise. The education component aims to educate people to understand pain, physical activity, healthy eating, drug management and simple ways to cope through self-management. Meanwhile, the exercise regimen is a progressive exercise programme and tailored according to the individual's abilities and needs including strength, balance, coordination, control and function. It is delivered twice a week for 6 weeks in small groups of 8-12 people (12 classes) (9, 10).

It is important to consider how the programme fits in with current practice and its acceptability and feasibility. This information is needed to inform future implementation if the trial demonstrates that the programme is effective for patients with knee OA. Thus, we conducted semi-structured interviews with patients with knee OA and their associated healthcare professionals. The goal of this study was to understand how OA is currently treated in primary care setting and to explore the perspectives of healthcare professionals and patients on the acceptability and feasibility of a structured self–management programme that involves community approach.

## Methods

### Design

A pragmatic qualitative approach was chosen for the study, whereby it does not adhere to any specific methodological approach, but is concerned with the real-world issues related to the stakeholder's perspectives of the ESCAPE-Pain programme, it, therefore, takes a more general approach to the area of study (12). The aim was to explore the beliefs and attitudes of healthcare professionals and patients with knee OA toward the ESCAPE-pain programme, including their perspectives of the current management of knee OA. The findings will inform the adaptation of ESCAPE-pain programme for implementation in the Malaysian healthcare context.

Face-to-face individual semi-structured interviews were undertaken to explore the overall opinions of the participants concerning their current management of knee OA and their perspectives of the ESCAPE-pain programme. The programme consists of education and exercise components were thoroughly explained to healthcare professionals and patients with knee OA by providing the programme booklet and exercise diagram. For the education component, they were asked about simple education, self-management, coping strategies, and exercise regimen. While the exercise components, they were asked about each exercises mode such as using static bike, bench, boards, theraband, and other physical movements. Interviews were chosen to obtain personal opinions that participants may not have been comfortable with sharing in the presence of other people, such as with focus groups (13). Interview guides for both healthcare professionals and patients were developed based on a review of the literature, the study context and the experience and knowledge of the author in the field (Table 1). This included questions considered relevant for eliciting pertinent information about how participants might engage with the programme. The interview guides were initially written in English and translated into Malay. The translated documents were piloted among Malay native speakers to check for understanding and clarity of the language. The semi-structured interviews were guided but at the same time, the interviewees were open to what participants say by conducting the interviews conversationally. The style of the conversation provided flexibility to probe or ask follow-up questions based on interviewees' responses (14). The ESCAPE-pain programme was introduced to the participants after asking about their experience of managing their knee OA.

**Table 1:** Example of interview guide for healthcare professionals.

**Topic**	**Specific objective**	**Example**
**Healthcare Professionals**		
Current management for patients with knee OA including support, facilities, effectiveness, role, barriers and influential factors.	To explore healthcare professionals' experiences, concerns and needs in the management of knee OA.	Could you explain about the support or facilities provided in this hospital to help patients in managing their knee OA?
Views on the suitability of education component and exercise regimen interventions from ESCAPE-pain programme.	To explore healthcare professionals' cultural and religious beliefs as regards the ESCAPE-pain programme.	Do you think that simple education will work for patients with knee OA? Why? Do you think that an exercise regimen for an individual will work for patients with knee OA? Why?
Recommendations of other relevant strategies, interventions and tools for consideration.	To consider how the ESCAPE-pain programme could be integrated into the care pathway in the Malaysian healthcare system.	Which other strategies, interventions or tools would you consider including as one of the elements? Please explain.
**Patients With Knee OA**		
Experience of self-management of knee OA.	To gain insight into patients' views regarding management of knee OA.	What is your opinion of the effectiveness of the service provided in helping you to manage your OA?
Views on treatment, facilities, effectiveness, barriers and influential factors of the hospital/healthcare.	To explore patients' experiences, concerns and needs in the management of knee OA.	What are the barriers/influential factors when receiving treatment from the hospital?
Views on the suitability of education components and exercise regimen interventions from the ESCAPE-pain programme.	To explore patients' cultural and religious beliefs about the ESCAPE-pain programme.	Do you think that simple education will work for you? Why? Do you think that a personalized exercise regimen will work for you? Why?
Recommendations of other relevant strategies, interventions and tools for consideration.	To consider how the ESCAPE-pain programme could be integrated into the care pathway in the Malaysian healthcare system.	Which other strategies, interventions or tools would you consider being one of the elements? Please explain.

### Participant Information

Participants were recruited from two selected hospitals in Malaysia. Healthcare professionals were included if they were one of the orthopedic surgeons, medical doctors, nurses, physiotherapists and occupational therapists with at least six months' working experience in orthopedics rehabilitation or with related qualifications such as Advanced Diploma in Orthopedics or equivalent; and directly involved in the management of patients with knee OA. A total of 14 healthcare professionals (eight men, six women) were approached and interviewed comprising two orthopedic surgeons, two medical officers, three nurses, three physiotherapists, three occupational therapists and one assistant medical officer (Table 2).

**Table 2:** Demographic data of healthcare professionals.

**Respondent**	**Gender**	**Age**	**Occupation (Highest qualification)**	**Work Experience in years**
HCP01	Male	32	Nurse (Masters)	8
HCP02	Female	36	Nurse (Advanced Diploma)	10
HCP03	Male	29	Physiotherapist (Diploma)	8
HCP04	Female	29	Physiotherapist (Diploma)	7
HCP05	Female	32	Occupational Therapist (Diploma)	10
HCP06	Female	33	Occupational Therapist (Diploma)	11
HCP07	Male	35	Occupational Therapist (Bachelor's Degree)	12
HCP08	Female	37	Nurse (Bachelor's Degree)	14
HCP09	Male	46	Assistant Medical Officer (Diploma)	19
HCP10	Male	33	Physiotherapist (Bachelor's Degree)	5
HCP11	Female	29	Medical Officer (Bachelor's Degree)	5
HCP12	Male	45	Consultant Orthopedic Surgeon (Masters)	25
HCP13	Male	29	Medical Officer (Bachelor's Degree)	5
HCP14	Male	34	Orthopedic Surgeon (Masters)	9

Patients were included if Diagnosed with OA affecting the knee by a medical officer based on CPG MOH (7), and mentally capable of giving informed consent; Aged 50 or above, the minimum requirement for the diagnosis of knee OA based on CPG MOH (7). Twenty patients were approached via clinic appointments and 18 completed the interview sessions. Two patients withdrew due to lack of interest and time constraints. The group consisted of both genders (male *n* = 5; female, *n* = 15) and ages ranged from 51 to 81 years old. The participants were from various ethnicities and cultural backgrounds (Malay, *n* = 9; Chinese, *n* = 8; Indian, *n* = 3). Seven participants had literacy problems and the rest had various levels of education; three were still working as civil servants at the date of the interview. The time since diagnosis with knee OA varied from 1 to 30 years (Table 3). Participants were excluded if they were unable to understand the Malay language.

**Table 3:** Demographic of patients with knee OA.

**Respondent**	**Gender**	**Age**	**Ethnicity**	**Level of educational background**	**Duration of knee OA in years**
P01	Female	68	Malay	No formal education	20
P02^*^	Female	71	Malay	No formal education	6
P03^*^	Female	70	Malay	No formal education	10
P04	Female	80	Chinese	No formal education	30
P05	Female	64	Malay	No formal education	6
P06	Female	61	Malay	MCE	4
P07	Male	56	Malay	Master's Degree^**^	7
P08	Female	74	Chinese	No formal education	12
P09	Female	61	Chinese	MCE	7
P10	Male	51	Chinese	MCE	3
P11	Female	64	Chinese	MCE	9
P12	Female	56	Malay	Diploma^**^	5
P13	Female	55	Chinese	LCE	5
P14	Female	69	Chinese	MCE	8
P15	Male	62	Malay	MCE	16
P16	Female	64	Indian	Standard Three, PS	25
P17	Female	64	Indian	LCE	4
P18	Female	70	Indian	Standard Six, PS	1
P19	Male	60	Malay	MCE	10
P20	Male	53	Chinese	Bachelor's degree^**^	1

* *Incomplete interview and withdrawn from the study*.

** *Still working. MCE, Malaysian Certificate of Education; LCE, Lower Certificate of Education; PS, Primary School*.

### Procedure

This study obtained approval from the University of Manchester Research Ethics Committee (Ref:16451) and the Medical Research Ethics Committee (MREC), Malaysia [Ref: (10)KKM/NIHSEC/P16-1552]. Hospital OA teams were identified to acquaint them with the aims and details of the study, and to solicit their authorization and co-operation.

Information was obtained about the members of the OA healthcare professionals' teams including representatives from primary care medicine, rheumatology, nursing, and physiotherapy staff such as gender, age, profession, and number of years in service. Healthcare professionals were purposively sampled by years of experience, age, and gender to reflect the diversity of the different healthcare professionals who support patients with OA. Purposive sampling provides rich information with diverse perspectives of the exploration of phenomena (15).

Meanwhile, patients diagnosed with knee OA were identified from their attendance at an outpatient clinic appointment, from outpatient clinic census or hospital records. A meeting was conducted with the members of the clinical staff regarding the study and criteria for patients. Patients were asked by the member of clinical staff if they were willing to be approached by the researcher. Potential healthcare professionals and patients were approached, provided with verbal information about the aim of the study, how the data would be conducted, what participation was required of them and the time needed for the interview before consent obtained. The interview sessions were audio-recorded and lasted between 60 and 90 min. All interviews were conducted in Malay language by the same researcher. The participants were informed about their right to withdraw from the study and confidentiality of the data.

### Analysis

The interviews were analyzed in Malay using QSR NVivo version 12 (16), to preserve the implicit and contextual semantics, and then translated into English. Issues of bilingualism were overcome by checking and re-checking the audio against the written transcriptions to ensure consistency. The process of transcription, anonymisation, and analysis using the framework approach generates themes through step-by-step contrasting and comparing across the data which produces highly structured findings (17). Framework analysis is beneficial by clearly tracking the movement of data (18, 19). It also follows well-defined procedures and provides the possibility of reconsideration and reworking ideas which enables collaborative analysis (19). Thus, the analytical stages lead to the final interpretation of the research findings (20).

Based on the transcripts, initial categories were developed. Then the initial themes were identified, and the connections between themes were analyzed to identify the main themes and subthemes. This process involves intuitive and logical thinking to judge the meaning and examine the links, to fully address the research questions (21). The initial themes were constructed based on the issues informed by the research objectives, and healthcare professionals' and patients' perspectives on the transcripts. This familiarization process continued until the information had been fully understood and all transcripts reviewed. Meetings with the research team were conducted to discuss the dataset and develop consensus of coding and interpretations.

## Results

Overall, the data reflect the healthcare professionals' experience in relation to the management of patients with knee OA, their explanations of how they cared for patients with knee OA, their perspectives of the ESCAPE-pain programme, and potential modifications that they think might be helpful for patients with knee OA in Malaysia. Both healthcare professionals and patients were positive toward the implementation of the ESCAPE-pain programme among the community. Positive views of the ESCAPE-pain programme were reported, with patients preferences and their understanding of the commitment required. The findings are summarized under three key themes: eagerness to know about ESCAPE-pain; coping with knee OA, and desired care. The main themes and subthemes that emerged from the data are outlined in Table 4.

**Table 4:** Themes and subthemes from interview data.

**Participants**	**Themes**	**Sub-themes**
Healthcare professionals	Positive views of the ESCAPE-pain programme	Current challenges create future opportunity Enabling the understanding of intervention through education Concerns about the exercise component
	Anticipating the preference of the patients	Possibility of participation Post-intervention Cultural aspects and beliefs
	Understanding of commitment	Family and social support Ability to adhere Motivational status
Patients with knee OA	Eagerness to know about ESCAPE-pain	Excited about the programme Ability to exercise
	Coping with knee OA	Difficulties of OA Complementary therapy in a cultural context Anxiety
	Desired care	Effective communication Continuous support

### Healthcare Professionals

#### Positive Views of the ESCAPE-Pain Programme

##### Current Challenges Create Future Opportunity

The healthcare professionals described their management of patients with knee OA and the provided services for these patients. The healthcare professionals understood their role in providing the best available treatment to patients with knee OA. Healthcare professionals described that in providing the services to patients with knee OA, they encountered many challenges such as shortages of staff, overcrowding of patients and high workload.

At the hospital, we have limited manpower to provide the best care to all patients… We try to educate them, but for various reasons, we cannot deliver the best… We don't even call them to ask about progress… The only thing is, we ask them during the next appointment visit… That's all… If you run education in a group like this programme (ESCAPE-pain), they will be happy… Meeting people could also make them happy… (HCP04)

We have time constraints looking after the patients while they're attending their appointments… Running the clinic effectively is added to this difficulty… It's everyday business, many patients… Every day… If you mentioned the programme could be conducted at home, that would be better… (HCP08)

These challenges were routinely observed in current practice. The ESCAPE-pain programme was described as being able to potentially facilitate the process of delivering healthcare services to patients with knee OA without requiring a visit to the hospital. The healthcare professionals agreed that recruitment to the ESCAPE-pain programme should be based on the availability of the patients and what the instructor considers to be a manageable number to support.

##### Enabling the Understanding of Intervention Through Education

The healthcare professionals described the component of education as a valuable addition to increase understanding regarding self-management of knee OA. They agreed to most of the content of the programme as described by the interviewer. They noted that in Malaysia there are different ethnic groups, different beliefs and different cultures. This understanding of the complexity of the Malaysian people is important as it may influence the implementation of a new programme and its acceptance. The healthcare professionals highlighted that the educational component in ESCAPE-pain could facilitate the understanding of the intervention among the Malaysian people.

When we teach them, we demonstrate the real thing for them; they would accept it… So far, they are tolerant… Through effective education, they will know what to do… (HCP03)

We should reinforce the programme with pamphlets, videos or a related website for their reference… I understand that those are emphasized in the ESCAPE-pain programme… Just check whether the patients involved have the access to those technologies mentioned… (HCP07)

All components in the ESCAPE-pain programme were viewed by the healthcare professionals as helpful for patients with knee OA. In addition to that, all of them spoke positively about the implementation of the ESCAPE-pain programme in Malaysia.

Implementing this (programme) will be very good. Most of the patients agree with getting health education from us. We tell them what to do based on their ability. They have no objection to doing it. The only thing is they might complain of pain. No excessive movement is what they prefer… (HCP02)

This (ESCAPE-pain programme) is good. We can give it a try. Call up a few patients who will agree to come to this session. It might help them… (HCP11)

The educational component of ESCAPE-pain consists of relevant topics that could facilitate the understanding of the intervention in the Malaysian context. The healthcare professionals highlighted that, in the context of Malaysian people, some older people attending hospital for treatment had not received formal education in their youth due to the political and economic difficulties during their childhood. Some of them had low levels of literacy but were keen on the use of step-by-step pictures (such as photos or illustrations) and numbering.

Some of them do not know the Malay language, we talk to their daughters… Some also do not know how to read: we provide them with illustrations… But sometimes, we don't have the resources to give to the patients… (HCP01)

Many of my patients attending for treatment do not know how to read the Malay language… And some of them cannot read at all… But they know numbers… Simple instructions might work… (HCP06)

A simpler version with more pictures was suggested, but the full version was found to be helpful for the family members and friends in understanding in advance the support needed.

##### Concerns About the Exercise Component

Referring to the exercise components in the ESCAPE-pain programme, the healthcare professionals tended to state that any equipment involved (particularly the static bike) may not be acceptable for the patients. Among the reasons were safety issues and the economic background of people suffering from knee OA in Malaysia. The exercises could be modified or replaced with another related activity, based on the capability of the patients.

Usually, we will advise the patients to use a water bottle (as a weight) to replace the quadriceps bench… There are different types of (muscle) movement, put the weight on a different side… (HCP03)

If they don't have a bike at home, we could advise them to walk if our aim is for cardio (endurance). If our aim is for their range of movement, we may advise them to cycle their legs while lying down… Just imagine how you pedal a bike… Or if they have very limited range, just swing the leg… If they have improved, they may continue on to a heel slide… (HCP04)

The alternative approaches provided by the healthcare professionals are not contradictory to the exercise regime in the ESCAPE-pain programme. This was checked with the originators of the ESCAPE-pain programme. Modifications can be made depending on the objective of the exercises either for endurance, strength, balance or flexibility. Meanwhile, the healthcare professionals perceived that some of the exercise types may not be suitable for older patients with knee OA as they could increase their risk of a fall. They suggested that a safety assessment must always be conducted.

Usually, we don't advise the patient to do a wall squat in the 90-degree position. That is dangerous. We need to stand by with a cushion or mattress (on the floor), just in case… They might fall (HCP07)

If the patient wants to try the boards, we must supervise. I am afraid they will fall. It will put pressure on the knee. The wall squat is also risky, putting an extra load on the knee… (HCP10)

Although exercises without sophisticated equipment are offered in the ESCAPE-pain, the main aim is to encourage the participants to engage with the exercise based on their capability, regardless of the type of exercise. Modifications must be true enough to the original ESCAPE-pain programme without jeopardizing the core components of the programme.

#### Anticipating the Preference of the Patients

##### Possibility of Participation

Although the components of ESCAPE-pain are relevant to the context, the healthcare professionals reported that there may be possible barriers to participation. The healthcare professionals gave an example of current treatments provided by the hospital. Some patients were reported to have a lack of motivation to follow the prescribed treatment. These patients are seen as not prioritizing their health due to other possible commitments such as being the main financial provider for the family.

For those who are doing business for a living, the only thing we can provide is education. For sure they have no time to do other things. They will be focusing more on their daily business. Different for a housewife: she might do this (exercise) while watching TV. At least she does… (HCP04)

Patients might default on the treatment if they feel that it is difficult for them to attend… As healthcare professionals, we want to see them, to look after them… They will only come to the hospital if they encounter any problems… (HCP14)

Some healthcare professionals perceived that some barriers to participation may be due to age-related factors that may affect the ability of some patients to perform the tasks related to the treatment. Some also reported that the patients' sedentary lifestyle had become a norm already, and thus difficult to change.

I think because they are old, the older people, they don't even have any desire to move. Sometimes OK, sometimes unmotivated (Malay: malas)… (to move)… They might not participate effectively… (HCP04)

They are old already to me. In addition, they have had no formal education before; they might be able to understand simple instruction only… It might be difficult to perform an effective intervention for them… (HCP11)

Other factors were pointed out by the healthcare professionals as a reason for the ineffectiveness of current treatment. Three main aspects were highlighted: long waiting lists, time constraints and oversubscribed services.

The gym (physiotherapy) is small, packed; the patients frustrated when they come. Also, it's difficult to park, the distance between one appointment to another is long… There's nobody around to bring them and fetch them from hospital… It's a logistics issue (HCP08)

If we have time to educate the patients during free clinic time, we will. The fact is that we have limited staff, so we must refer them to physiotherapy… We hope they may have time to do it… (HCP13)

Thus, understanding the difficulties of delivery of treatment to patients with knee OA may facilitate the understanding of possible participation in the ESCAPE-pain programme. ESCAPE-pain was seen as best implemented in the community or home setting due to current difficulties faced by both healthcare professionals and patients with knee OA in getting the best treatment at the hospital.

#### Post-intervention

Another theme highlighted by the healthcare professionals was the continuous engagement after the completion of the programme. Assessing the capability of the patients and understanding the socio-demographic differences were seen as important for post-intervention. Apart from different cultures, the Malaysian community also has differences in economic status and geographically different areas such as urban, suburban and rural areas. These factors may influence patients in terms of access to healthcare facilities, recreational and daily needs. Pre-assessment of patients' backgrounds may help to identify the best strategies for an individualized exercise provision.

We immediately educate them about home exercise… Every appointment, we will teach the patient some home exercise… We will tell them what they can do, what they cannot do…What they should do…What to be cautious of… (HCP10)

The patients might not be able to afford to buy the equipment. Those attending the government hospital are usually low to middle income people (HCP13)

The healthcare professionals expressed that different ethnic groups also practice different daily living activities based on their health perceptions. It was observed that some ethnic groups may practice better physical activities than others.

Malay especially, and the older females, are not keen to do exercise: they see it as something strange to them… (HCP02)

To me, the Chinese are more disciplined in terms of performing the exercises; they have Tai Chi, even group dancing for example, but not Malay… (HCP03)

Therefore, understanding the differences between the ethnicities may be helpful when considering how to present or implement the programme. The healthcare professionals also perceived that the patients may require continuous support with good communication skills in order to help them to feel positive about the programme in the home setting.

Some patients are willing to buy home exercise equipment such as a static bike… They will use it initially… Toward the end, they might not do it again… The home environment is sometimes not encouraging… If they are really enthusiastic, yes, they will do it… Otherwise, they won't do it… Home-based exercise should be encouraged… (HCP03)

The delivery of the programme could be modified according to the patients' beliefs and daily living activities. This may help the patients continue to engage with the programme after completion of the intervention.

##### Cultural Aspects and Belief

The majority of Malaysian people embrace religious beliefs which they practice as part of their activities of daily living. They perform rituals or prayers based on their faith and these fulfill the needs of a follower. Therefore, if they have difficulty in performing these activities, they may seek help by searching for any available treatment to solve their problems. This was also highlighted by the healthcare professionals: that the patients who come to hospital need treatment to improve their daily living activities including performing their religious activities.

When they pray, they will have a problem. So they come to get our advice. If they are not confident in us, we will get the religious officer to teach them how to pray while sitting. They still can do it even though they feel that it is not perfect (HCP05)

We advise the Muslims particularly to pray sitting… However, there is not much difference… Before attending hospital they also prayed sitting… The only thing is that they will feel more confident when the healthcare professionals advise them according to their beliefs… (HCP11)

Thus, restrictions in performing religious activities appeared to be a potential motivator for the patients to seek available treatment. An alternative method of treatment for symptoms may be perceived as a positive alternative by older people. When providing education, simple instructions were seen as helpful for older people. In these circumstances, the delivery of the programme is illustration based, with active group discussion and follow-up strategies to enhance understanding.

Other than undertaking physical activities, the healthcare professionals perceived that the patients with knee OA may have their way of managing their symptoms through traditional practices. The healthcare professionals reported having no problem with the patients' traditional approach as long as it did not worsen the patients' symptoms.

Sometimes they will see a “bomoh” (traditional healer) first, and they will only come to us if it does not work with them… We advise them to come to us first, but then that is their belief, we cannot force them (to come to the hospital) … (HCP06)

Many of them will go for massage, hot compress using hot stones (Malay: bertungku), but… Hmmm… We cannot prevent them because it is a norm here… If you have pain you go for a massage, or hot compress… (HCP13)

While reading through the ESCAPE-pain programme, the healthcare professionals highlighted the need to comply with the local context for some of the contents. They believed that this would not change the main content of the programme.

In my experience, most people… Particularly the older people… Are sensitive to cultures in many ways… The food, the lifestyle, the relationships and so on… (HCP09)

I would recommend that the programme uses local photos or pictures, portraying our way of life, which could be practical for (the older) people to follow… (HCP14)

Therefore, incorporating cultural aspects and beliefs may facilitate the understanding of the preferences of patients with knee OA. This cultural knowledge may facilitate the process of adapting the programme into the Malaysian context. The recommendations of the healthcare professionals regarding the culturally sensitive content were used for the adaptation of the ESCAPE-pain programme in this study.

#### Understanding of Commitment

##### Family and Social Support

The healthcare professionals indicated that there may be some positive factors that may facilitate concordance with treatment. The influence of caregivers including family members and their level of education contributed to positive reinforcement. The healthcare professionals also emphasized that caregivers may also influence the decisions made by the patients regarding treatment.

We try to use the motivational interview in our care… We ask them to describe what they would do with the family if they were healthy… We try to motivate the family as well to care for the patients… (HCP07)

We have to see them with the caregiver, the spouse, son or daughter; they can be involved together (HCP05)

So, the opportunity for healthcare professionals to involve family members or caregivers in treatment was perceived to be important. This factor may assist with the acceptance of the programme by patients. Social support was reported to be important in increasing engagement with any treatment. The patients seemed to have good peer support when socializing with other patients with similar conditions.

Usually, the patients will be anxious at the beginning… However, when other patients explain the management and care to them, it influences them … Supporting each other is seen to be effective… They need to socialize at the beginning of the treatment… (HCP06)

When we conducted the sharing session, we found the patients' self-esteem was better if they managed themselves… Unlike previously, they feel loneliness… They know that other people also have similar problems to them… We noticed that they tend to attend appointments… Because of other people similar to them… Better adherence, I believe… (HCP07)

Thus, for patients with knee OA, being supported by relatives and other patients with similar conditions appears to facilitate the acceptance of treatment or intervention of care.

##### Ability to Adhere

Another concern of the healthcare professionals was the ability of prospective participants in the programme to adhere to the twice-weekly schedule for 6 weeks. They highlighted that good communication, continuous reminders and building good rapport may be important to promote adherence to treatment.

If we provide the patients with systematic health education, I think they will follow it… It helps patients… Any types of diseases or problems, we provide health education… The most important thing is effective health education… (HCP01)

The patients will do the exercise intervention… But we need to remind them of those exercises… Follow-up calls are very helpful… We call the patients or their family or relatives just in case if they have any difficulties or problems… (HCP02)

The healthcare professionals added that the patients may also adhere if the programme suited their daily activities, though effective communication and gentle reminders were recommended.

I think we must keep telling (reminding) the older (people) generation… Only then will they keep following it (the content) … But… It depends on their acceptance as well… (HCP11)

Usually, when we advise them, we should follow it up (for effective education) … It sounds additional effort to do… But sometimes we have no time… Although we know it is essential to track the progress (HCP13)

The healthcare professionals added that, in their experience, regular reminders may help the implementation to be effective in terms of adherence to the programme. Thus, follow-up sessions were one of the important reported strategies that may assist with continuing engagement.

##### Motivational Status

According to the healthcare professionals, the educational status of the patients may also influence the acceptance of treatment or health-related education as the majority of the patients receiving treatment in the general hospital are people from low to middle socioeconomic class. Also, people from different ethnic groups may not fully comprehend the official language of Malaysia due to limited use of their own language, such as Mandarin, Cantonese, Tamil, or local dialects. These aspects could influence their motivational status in receiving care.

To me, education and practice, it depends on the level of education. If low, they will listen but not do. If they are educated people, they will listen and follow instructions (HCP06)

There are patients who are highly motivated to come to do exercise… This type of patient could support the other patients to do it together… The enthusiasm is really good to encourage other people (to do exercise)… (HCP08)

Motivational status was perceived to be one of the factors to consider to ensure a good outcome for the programme. Motivational levels could increase or decrease, but the most important was to keep motivating the patients through different methods.

### Patients With Knee OA

#### Eagerness to Know About ESCAPE-Pain

##### Excited About the Programme

While the ESCAPE-pain programme was being explained to patients with knee OA during the interviews, most of the patients expressed positive attitudes toward the programme. They were interested in getting further information about the programme and wished to be able to join the programme once implemented. They were excited about socializing with other people during the group discussion of the educational components.

I would seriously consider joining the programme. At least I can socialize with people with the same thing in common… (P08)

I have to take care of myself. I am not only living with knee pain, but also I am a breast cancer survivor. I do not want to be free (to have free time). I always tell myself, I will mark the calendar if I want to do something, and I think I can manage myself (P11)

They appeared to be pleased about the possibility of joining the programme. They perceived that the two components in the ESCAPE-pain programme may benefit them in managing their knee OA. They were also excited about self-managing their symptoms.

##### Ability to Exercise

The patients felt that most of the exercises in the ESCAPE-pain programme would be easy to perform and require little guidance for implementation. Some of the patients said that they would perform physical activities as part of their daily lives.

Sometimes when I lie down alone, I raise my leg… In supine position, I feel good. If I feel pain, I stop. If I sit on a chair, I will raise my leg up to 50 (times). I stop. I feel light (better) for a while… (P06)

I just walk every day. I just do hand movements, like this, like that, up and down. Sometimes I move my leg like this. I exercise myself. Nobody taught me… (P14)

The patients also mentioned that an exercise bike may not be affordable for them, while some exercises in the regime may also not be suitable for them as they had specific difficulties.

I feel nervous when I step down. Very painful (step down). A higher step is more painful. To step forward is very painful… It is very exhausting… I can't even run. Only walk… I can walk halfway and stop… (P07)

Squatting is very painful … If I want to stand, it is very painful… It is very stiff… I have to hold on to something to get up from squatting or sitting … (P09)

Thus, the exercise may need to be based on the participants' functional abilities as recommended in the ESCAPE-pain programme. Any activities which are painful for a given participant are discouraged in the programme. Only the equipment which is available for the participant is encouraged, if there is no equipment, the participants may complete other exercises. Some patients added that their daily practice of spiritual activities may also have a positive impact on them. This may help the patients engage with physical activities.

Usually, after morning prayer (subuh), I will do some exercises. The daily practice of prayer reminds me to do exercise. In the afternoon, if I have nothing to do, I will do exercise too… (P05)

In those days I went for exercise, did qigong, very active… Now, I wake up at 5 am, do some prayer, then I do yoga. Every day… I just started… Due to the knee pain. Now I can fold the leg. If I don't exercise, I am afraid it will jam… (P17)

Therefore, participants may need to be asked about the best time for them to practice exercise. Spiritual or religious practice may be part of the assessment as it may be considered the best time to include the exercise regime.

### Coping With Knee OA

#### Difficulties of OA

The patients revealed that they were having problems undertaking prescribed treatment by the hospitals for reasons such as family matters, time constraints, economic factors and lack of motivational support.

I have to wait for 2 to 3 h. Moreover, I am having difficulty communicating in good Malay language with the others. I feel so quiet… (P08)

If I go to the hospital, I will get free service. If I want to buy it, it is not affordable. It (the equipment) is very expensive (P16)

Usually, the treatment in the hospital is once a week. I think it is inadequate; otherwise, I have my own equipment. Meanwhile, I think that they (healthcare professionals) do not provide individualized treatment… That's my comment (P20)

However, they required treatment to manage their symptoms, which many Muslim patients mentioned affected their religious practice. They reported certain limitations.

If I want to pray, I must sit for a long period first then can only walk to the bathroom If it's morning prayer (subuh), it is not too bad to pray although I have to move my leg first before getting up. The reason for not going to the mosque is knee pain. If nobody takes me, I don't go. (P05)

I am having difficulty praying. I should sit on the chair to pray. I feel that it is not the perfect way of praying. I hope God will accept my prayer. (P06)

Participants reported that they wanted to overcome their symptoms in order to make themselves feel like better people through being able to perform religious activities. This factor appeared to be a good motivator for the participant to take part in an exercise programme. Either they had to go to the hospital, or they had to find alternative ways. They perceived that through education and exercise, they could perform the exercises by themselves.

The patients with knee OA also revealed that they faced lots of difficulties in daily life due to the disease. They believed that biological factors (age) were predetermined and therefore inevitable. However, the psychological factors (fear of exercise, low self-esteem) could be considered and controlled as a means of overcoming the destructive emotions if the treatment was suitable.

Everything is stiffening already. I am too shy to start doing exercises, I am too old. There's nothing much we can do (P08)

If I were having extreme knee pain, I wouldn't look for this (exercise) equipment. I have to accept that, it is from God. The time has come, the number of age has increased… (P12)

The patients expressed feelings of shame and inconvenience about doing exercises. They were afraid of what people around might think, they were not motivated, and also feared the risk of falling.

If I go to exercise, it is only because of knee pain. We are afraid that another illness will come. Maybe a fall. So, I think if safety precautions are taken to prevent any injuries, for me this will be better… (P12)

First thing, it is unmotivated (Malay: malas), the second thing is it is an inconvenience. Otherwise, if there are not too many types (of exercises) then it might be OK. I prefer cycling…So I am seriously thinking of going to the gym (gymnasium). (P20)

Recognizing the difficulties of knee OA and its consequences may enable facilitators to plan future interventions by using the facilitators and including activities that may decrease or overcome the barriers. Motivational interviewing as in the ESCAPE-pain programme seems to be a good activity to conduct during a group discussion in order to encourage behavioral changes among older people.

##### Complementary Therapy in a Cultural Context

The patients revealed that restriction of activities and constant pain are part of their difficulties of living with knee OA. They said that one of the strategies for managing symptoms of knee OA is the use of alternative or complementary treatment. Many different approaches were reported to minimize or decrease the burdensome symptoms, including Islamic traditional healing, massage, traditional Chinese medicine and thermal bed.

I have tried almost everything… Massaging… Few times I tried…After massaging I feel better…Sometimes I use hot stone compress (Malay: bertungku)… Like a postnatal mother… I also have tried the Islamic healing treatment (Alternative treatment using religious beliefs of practitioners: such as the use of the holy verses of the Quran and pray. The practitioners acknowledge that any diseases and healing processes are from the Creator. No pharmacological component)… (P15)

Initially, I felt very strong pain, I went for massage… For 2 weeks of massage… Because I want to cook, nobody would cook for me… Massaging also never heals… I have tried the free promotional Ceragem (automatic therapeutic thermal massage beds) for almost one and a half months already now (P16)

Sometimes I go for a massage…I have tried a Chinese traditional practitioner (Malay: Tabib cina), acupuncture… almost everything - whatever people said… (P19)

The interviews revealed that the patients received treatment at the hospital as well as trying different traditional and complementary therapies to improve their conditions. The ESCAPE-pain programme offers few alternatives to exercise for the patients to decrease the symptoms and promote wellbeing.

##### Anxiety

The patients also highlighted that they were worried about their current condition. They appeared to have a low level of understanding of how to cope with anxiety. Their activities of daily living had also changed due to the knee OA. Some of the patients were unable to read or write, including the education materials. Unlike patients with a good educational background, they appeared less able to find ways of coping with their concerns.

I just keep quiet when the healthcare professionals talk, I can't read. Do not read the book (Al-quran), anything—cannot read. I have been working from a young age… You demonstrate to us first, then we can do it. Provide us with instructions using pictures and numbers, we will follow… (P05)

I cannot read at all. If you provide me with a picture and number, I think I could… (P01)

I will only do whatever I think good… Other people do not suggest anything to me… probably I don't ask them (laugh)… I will search for further information if I want to know…(P20)

Although they reported that healthcare professionals had given them education pertaining to the care of knee OA, they stated that they just kept quiet if they did not understand. However, some of the patients suggested that using numbering methods with a picture or step-by-step diagram may help.

#### Desired Care

##### Effective Communication and Approach

The patients appeared to prefer the healthcare professionals to talk to them by giving advice including strategies to manage their symptoms. Two-way communication in educating people with knee OA was suggested for better understanding and acceptance.

Sometimes I fell asleep when the nurse was giving health education (feeling bored). Sometimes, I will tell the nurse whom I know, that if everybody follows the advice, nobody will go for surgery (knee)… (P06)

Healthcare professionals should say something advisory. They must communicate with the patients at every session attended. It will improve the acceptance of information given… I received the information about doing this and that… But I was not asked whether I could do that or not… (P20)

Thus, two-way communication was repeatedly suggested to encourage patients to actively participate during group discussion. It might help them accept the content to practice in their daily activities. Patients preferred polite communication such as simply asking about their condition.

I don't want people (healthcare professionals) to ask so many questions… Just ask “how are you?”…“Do you feel better today?”… Or “Is it good for you?”… Things like that… Nothing more is expected from me… (P08)

I prefer staff that can communicate with me… To show they're compassionate about me… Communication is very good. If they could do that, I would feel happy… (P11)

Patients requested that every person should be treated individually based on their condition. Every person may have a different problem and ability. The way healthcare professionals approached them was deemed important.

We are old already, thus we tend to be sensitive… Although something simple also, we take it seriously… We become too bad… It's sensitive (P01)

If people gently talk about the management of care, we tend to accept it… Sometimes when we are also not in a good mood, it changes because of the way people communicate and approach us… (P15)

The patients appeared very positive about accepting any information related to the management of care based on the way people communicated and approached them.

##### Continuous Support

Although some patients appeared aware that their motivational levels may decrease with the implementation of a self-management programme, they suggested a few things to maintain engagement with the programme. Most of the patients were excited that the ESCAPE-pain programme includes group discussion. They believed that by socializing with other people, their motivation level would increase. With the support of a facilitator, they thought that they may follow the flow of the programme.

If we have friends, we will see other people with the same fate, we will be motivated; we might not need a constant guide, and friends also can help (boost motivation)… If a nurse or a doctor said to me, I would follow. We may need someone to tell us what to do, what the consequences are. If we do it ourselves, we do not know what will happen… (P07)

If I do it myself, I will do something that I know. I can't do all the exercises. I need guidance from people (healthcare professionals) to do it… (P17)

As the programme is aimed at patients with knee OA, it is important to consider their suggestions. A few areas were suggested for integration into the programme, including effective communication and motivational support. Motivation to be involved in treatment or any related programme relies on several factors. The patients informed that they were much unmotivated to exercise.

It takes time… That makes things difficult. If I need to, only then I will do it. If not, I won't do it. I will bear (Malay: tahan) the consequences (of the disease) until it becomes severe enough to look for treatment. I believe our attitude must change… (P07)

After that, I feel unmotivated (Malay: malas), too much work to do. If there is no work, it would be fine. Put it in front of the TV (the exercise equipment), just temporarily use the equipment for exercise. Furthermore, we are getting older, unmotivated (Malay: malas); the most important thing is that we're unmotivated (Malay: malas) … (P12)

Some of the patients also reported that they needed attention or continuous guidance from healthcare professionals to do the exercises or follow the treatment regimen.

In case we miss the session, call for follow-up. The staff (healthcare professionals) should do this I think… We are the countryside people (Malay: orang kampung). If we don't tell them, they won't. People like me may think, if we have guidance, we will do it; if motivated, we do; if not, we don't. (P07)

If someone guides me in doing all those things it would be better. Sometimes, if we want to do it, it might be wrong. Maybe if a doctor or nurse could correct it, it would be better. (P09)

Some of the patients also revealed that they felt very positive about continuing any treatment or programme or exercise if it made them feel better. They believed that by completing exercise, they may get some benefit.

If I feel better, I will continue to do it. If there are no changes (or it becomes worse), I will stop doing it (P08)

My father was a yoga master… I do yoga every day… Just started because of the leg (knee)… I practice it, now I can bend the knee… If no exercise, I am afraid it will be jammed (stiff)… (P17)

Some patients mentioned that if family members supported them, it may influence the way they practice at home.

I live alone. So will be unmotivated (Malay: malas) to do it. If my children are around, I feel different. Let them observe what we are doing. My children like anything related to health. They always remind me, like 'have you done it?'… If they are not around, I feel unmotivated. They remind me because they know I am in pain. (P06)

I don't know how to do it. I feel unmotivated (Malay: malas) at home, cannot do it… (laughing) Someone has to force me: only then it's OK. (P13)

These findings suggest that many factors may influence patients' motivation, including healthcare professionals, family members and activity-related outcomes. Each patient may have different motivational factors that may help them adhere to the programme. Taking these factors into consideration may be very important.

## Discussion

This study aimed to explore the perspectives of healthcare professionals and patients with knee OA in managing knee OA and the ESCAPE-pain programme. The findings could be used to inform cultural modifications to the ESCAPE-pain programme. Some considerations were identified from both groups: views of current management; experience, concern and needs; cultural influences; attitudes toward the ESCAPE-pain programme.

### Views of the Current Management of Knee OA

Both healthcare professionals and patients with knee OA agreed that current management of knee OA in the hospital is good but access is problematic due lack of staff, overcrowding, long waiting lists, personal matters and time constraints. The distance of the facilities from their homes, transportation issues, and the cost of traveling were also considered burdensome. These problems are also reported in other parts of the world (22, 23). Thus the difficulties faced by both parties may require an effective self-management intervention outside the healthcare facilities, possibly in a community setting (24).

Furthermore, communication barriers were also reported by healthcare professionals and patients. Lack of two-way communication and difficulties in understanding instructions were among the issues (25). In another study, some patients were reported to be reluctant to discuss any therapy undertaken with their healthcare professionals (26). They may not have been comfortable discussing these issues with their healthcare professionals. The interviewees suggested that interactions between the patients and healthcare professionals could improve adherence to the treatment and keep the patients engaged with the programme (27).

Some of the patients also reported having literacy problems which could interfere with receiving effective treatment when using reading materials related to the treatment. Although the healthcare professionals mentioned that home exercise was prescribed for the patients, it was not beneficial for some of the patients, possibly due to these reasons. It possibly shows that a breakdown of communication had occurred. Thus, an improvement in communication skills between the healthcare professionals and patients is needed, which could create a positive effect on the process of rehabilitation including improving the quality of patients' life (28).

An alternative that is suggested is motivational interviewing during group discussion; it is used as a platform to encourage a behavioral change in older people (29). Through discussion, it provides the opportunity for the participants to share their views with other participants, promoting two-way communication. Personnel providing rehabilitation services also could include strategies to cope with the intervention programme, for instance, individualized strategies for self-managing the symptoms (30), as expected by the patients (31). The descriptions of current management have identified barriers to managing patients with knee OA. Thus, possible solutions were identified before adapting the programme to the context for implementation.

### Experiences, Concerns, and Needs

The patients said their experience of having knee OA has restricted their daily activities. Patients also stated that their pain worsened sometimes in the day when doing activities or even small movements. They also reported problems with being able to squat. This could lead to stressful situations and impact their quality of life (30).

Lack of motivation is another contributing factor highlighted by both groups of participants. The participants reported that they are already too old to do exercise (32, 33), accepting the societal norm of having a sedentary lifestyle. They also felt fear of doing exercises, shame, and fear of making a bad impression on their community and family. Motivation was very important if patients are to participate in the programme. Some of the patients also believed they were quite powerless to do anything other than what they actually did, in a fatalistic way (34). Some of them also accepted the cause of pain, without complaining about it, and were afraid to do further exercise for fear of causing pain (35). These factors could contribute to their low levels of motivation.

The patients also needed support from healthcare professionals, family, and society in terms of an understanding of the differences in sociodemographics, activities of daily living, and the practice of different ethnic groups' living activities. The success of an intervention programme seemed to be determined by continuous support from the family and community (36). The patients required continuous support from the family members, friends, or other people with the same problem as them. Continuously supporting the patients was seen to be important, through various techniques including education, advice, counseling, and guidance (37). Continuous encouragement from the staff and the involvement of family members and friends may have kept the patients engaged with the programme. Increased engagement provided opportunities for patients to ask questions about health promotion and disease prevention (38). Healthcare professionals needed to personalize the advice, including support for the individual's needs, according to their current symptoms and their consequences on their activities of daily living (39). Understanding the patients' experience, concern and needs may have increased the acceptability of the programme within the context.

### Cultural Influences

Culture is the platform that we engage with in everyday life, and is not a concrete thing (40). It includes the language, relationships, behaviors, and shared values of the way we live. Culture could influence how patients with knee OA perceive disease and could provide the opportunity for them to look for treatment if they have difficulties in fulfilling their cultural needs. Patients reported that they have problems practicing their religion in daily life. The healthcare professionals agreed when the patients asked them for advice related to cultural needs.

Both types of participant also reported that various techniques were used to alleviate the symptoms of knee OA, particularly pain, using locally commonplace alternative and complementary therapies. Massage, herbal and cultural-based treatments (such as Islamic medical treatment, and traditional Chinese healers) were among the options. In another study, it was reported that the use of chiropractors, massage and dietary supplements were among the most widely used complementary or alternative modalities (26). Besides, herbs and topical ointments were also found to be commonly used traditional medicine among the Malaysian population (41). Within the ethnic groups also, people use traditional medicine, reflecting various cultural practices across the community (42). They believe that traditional medicine containing natural materials from plants is less harmful and probably more effective than conventional medicine (41). These approaches may be worth integrating into the discussion to relate to their daily living activities. It may be worthwhile for healthcare professionals to be familiarized with the common traditional and complementary medicines taken by the patients (41). The instructor for the ESCAPE-pain programme may also make a further assessment of the traditional remedies options before guiding the patients regarding the use of a traditional or complementary approach based on current knowledge.

Meanwhile, patients reported that they were concerned about their spiritual needs. The majority of Malaysian people embrace one religion, which is in line with findings that more than 80% of the world's population associate themselves with a type of religion (43). Patients reported that while praying, whatever the religion, they could be in a state of relaxation. The relationship between religions and effects in life are variously reported and happen throughout people's lives (44). Although it is a personal issue in someone's life, it also has something to do with daily living activities. Patients value it for empowerment, peace, hope, and their faith (45). Difficulties in fulfilling spiritual needs may be a good motivator to look for an alternative treatment, which requires strong faith to create positive change. For some people, the perceptions of diseases have been influenced by their belief in life after death, consequently affecting their decisions about their condition (46). Thus, it is worth discussing the strength of their faith to create a positive change toward better management of knee OA.

Another related cultural difference in the programme is between dietary intake in the UK (western) and local foods. In Malaysia, as a Muslim dominated country, alcohol is not commonly used by the majority of people. However, it is taken by followers of some other religions. In practice, the healthcare professionals generally advise patients against alcohol consumption (7). Understanding a culturally sensitive diet has proven to be important for an intervention programme (36). The selection of food behavior is also strongly linked with the culture around good physical health (47). Meanwhile, other daily activities of older people in Malaysia could be different from other parts of the world. Some patients felt that undertaking exercise was strange to them at their later stage of life, but this was viewed differently in other parts of the world (48). Thus, understanding cultural activities in the society could tailor specific action goals to promote the acceptability of the programme.

### Attitude Toward ESCAPE-Pain Programme

Both healthcare professionals and patients with knee OA have positive attitudes toward the ESCAPE-pain programme, both education and exercise components equally. The programme was also reported by the patients as being a platform for them to socialize with other people, which had been reduced due to their knee OA (34). The education component was perceived to be a comprehensive package for people with knee OA. However, some of the older people in Malaysia have literacy issues, and illustration-based information is suggested for better understanding. Using pictures in educating the participants was reported to increase their attention, ability, ease of remembering and tendency to adhere to the education programme (49, 50).

The list of exercises in the exercise component was seen as appropriate to practice, similar to other studies findings regarding exercise for OA (34, 35). However, exercises using any equipment such as static bike may not be feasible due to financial constraints. Using a recreation or exercise bike is hardly seen among the elder people in Malaysia. Those who could afford to buy equipment were encouraged to do so. Some of the healthcare professionals and patients had negative attitudes toward the use of rocker boards, wobble boards and wall squats due to safety reasons, but the majority agreed that they could be used with the recommended safety measures.

To increase safety while performing a wall squat, adding a ball between the wall and the patient was suggested. One-leg standing was suggested as an alternative to board exercises (51). Safety is the utmost priority, particularly when dealing with older people. There are many risks, such as falling and injury. Therefore, the healthcare professionals advised that rocker boards and wobble boards should not be used in this study and they were replaced with one-leg standing. Meanwhile, the ability to perform a wall squat should be assessed for all participants with the highest safety consideration. Thus, modifications of the exercise programme based on patients' ability and acceptance of the context were encouraged in the ESCAPE-pain programme, to promote engagement with the exercise.

As for the delivery of the programme, it may be worth looking at the barriers faced by the healthcare professionals and patients with knee OA, provided with possible solutions in the delivery of the programme in the context. To keep people engaged in the exercises, assessment of their daily routine may help to identify the best time to perform the exercises. Based on the flexibility of the participants' and the instructor's time, sessions were arranged to suit the best time for both parties. The success of the intervention appeared to be facilitated by support from family and social, cultural sensitivity, and the appropriate level of literacy among the participants (36). The findings from both healthcare professionals and patients with knee OA concerning the possible integration of ESCAPE-pain will be modified into the ESCAPE-pain programme.

The strengths of this study include the use of appropriate design; translated interview guide was piloted; approaching two hospitals; patients were from various cultural backgrounds; recorded interviews and conducted by a native speaker. The qualitative approach was designed to gain perspectives on the ESCAPE-pain programme is seen as a valuable introduction to the healthcare professionals and patients with knee OA before adapting and testing the ESCAPE-pain programme. The reliability of this qualitative approach was also ensured through audio-recording, saturation and reflexivity (52). However, the limitation of this study could be the opposite gender of the interviewer may influence participants from sharing their experiences.

In conclusion, the present study aimed to elicit healthcare professionals' and patients' views about the ESCAPE-pain programme for implementation among the Malaysian population. The findings would be the platform for integrating and incorporating the ESCAPE-pain programme into the context with modification. Overall, the ESCAPE-pain was perceived as feasible and acceptable for implementation in Malaysian health care context. The integration of education and exercise components may be influenced by the community and the available resources. Possible modifications included: providing close contact with the older people with knee OA; approachable healthcare professionals; comprehensible modules; translations of language and lifestyle. Other techniques of programme delivery were suggested to engage patients, introduce the intervention and illustrate its proposed mechanisms more clearly during the next stage.

## Data Availability Statement

The original contributions presented in the study are included in the article/supplementary materials, further inquiries can be directed to the corresponding author/s.

## Ethics Statement

The studies involving human participants were reviewed and approved by University of Manchester Research Ethics Committee (Ref:16451) and the Medical Research Ethics Committee (MREC), Malaysia (Ref: (10)KKM/NIHSEC/P16-1552). The patients/participants provided their written informed consent to participate in this study.

## Author Contributions

All three authors involved in the design, developmental, analysis, and discussion of the study. The corresponding author involved in data collection.

## Conflict of Interest

The authors declare that the research was conducted in the absence of any commercial or financial relationships that could be construed as a potential conflict of interest.
